# Tropical origins of the record-breaking 2020 summer rainfall extremes in East Asia

**DOI:** 10.1038/s41598-022-09297-4

**Published:** 2022-03-30

**Authors:** Sunyong Kim, Jae-Heung Park, Jong-Seong Kug

**Affiliations:** 1 Physical Oceanography Department, Woods Hole Oceanographic Institution, Falmouth, USA; 2 Division of Environmental Science and Engineering, Pohang University of Science and Technology (POSTECH), Pohang, South Korea

**Keywords:** Climate sciences, Hydrology

## Abstract

The East Asian countries have experienced heavy rainfalls in boreal summer 2020. Here, we investigate the dynamical processes driving the rainfall extremes in East Asia during July and August. The Indian Ocean basin warming in June can be responsible for the anticyclonic anomalies in the western North Pacific (WNP), which modulate the zonally-elongated rainfalls in East Asia during July through an atmospheric Rossby wave train. In August, the East Asian rainfall increase is also related to the anticyclonic anomalies in the subtropical WNP, although it is located further north. The north tropical Atlantic warming in June partly contributes to the subtropical WNP rainfall decrease in August through a subtropical teleconnection. Then the subtropical WNP rainfall decrease drives the local anticyclonic anomalies that cause the rainfall increase in East Asia during August. The tropical Indian Ocean anomalously warmed in June and the subtropical WNP rainfall decreased in August 2020, which played a role in modulating the WNP anticyclonic anomalies. Therefore, the record-breaking rainfall extremes in East Asia that occurred during summer 2020 can be explained by the teleconnections associated with the tropical origins among the Indian, Pacific, and Atlantic Oceans and their interbasin interactions.

## Introduction

The East Asian summer monsoon (EASM) is one of the most active monsoon systems, affecting the weather and climate in East Asia and of great socio-economic importance for densely populated regions. Up to two-thirds of the annual precipitation occurs during summer over East Asia, and more than 40% of the summer rainfall falls during the rainy season^1–3^. In this season, the East Asian rainfall is often derived from tropical cyclone activity, the western North Pacific anticyclonic circulation (WNPAC), and a zonally elongated rainband referred to as Meiyu in China, Changma in Korea, and Baiu in Japan^4,5^. The understanding of the East Asian summer rainfall remains an outstanding challenge mainly because the East Asian region is influenced by complex interactions between tropical, and mid-high latitude systems^6^.

Studies on the mechanism of East Asian climate suggested that the WNPAC acts as an atmospheric bridge linking tropical forcing to East Asia^7–13^. It has been widely recognized that the WNPAC plays a key role in connecting the El Niño-Southern Oscillation (ENSO) to East Asia^8,14–20^. Although the ENSO is known as a major driver for East Asian summer climate, the tropical Indian Ocean also contributes to the development and persistence of the WNPAC^21–25^ during post-El Niño summers. Additionally, the relationship between the Indian Ocean SST and East Asian summer variability has been reported^26–32^.

It is suggested that the warm Indian Ocean sea surface temperatures (SSTs) during post-El Niño summers can contribute to the interannual variability of the EASM^23^. The tropical Indian Ocean warming causes the tropospheric heating via moist adiabatic adjustment^33^, which excites baroclinic Kelvin waves into the western Pacific inducing surface Ekman convergence on, and divergence off, the equator. The resultant suppressed convection and anticyclonic anomalies in the WNP may be associated with changes in the strength of the EASM. This process is known as the Indo-western Pacific Ocean capacitor (IPOC) mode, linking the tropical Indian Ocean and WNPAC in post-El Niño summer^23^. The WNPAC further affects East Asia through an atmospheric Rossby wave train with meridional dipoles in the lower troposphere, the so-called Pacific–Japan (PJ) pattern^14^.

**Figure 1:**
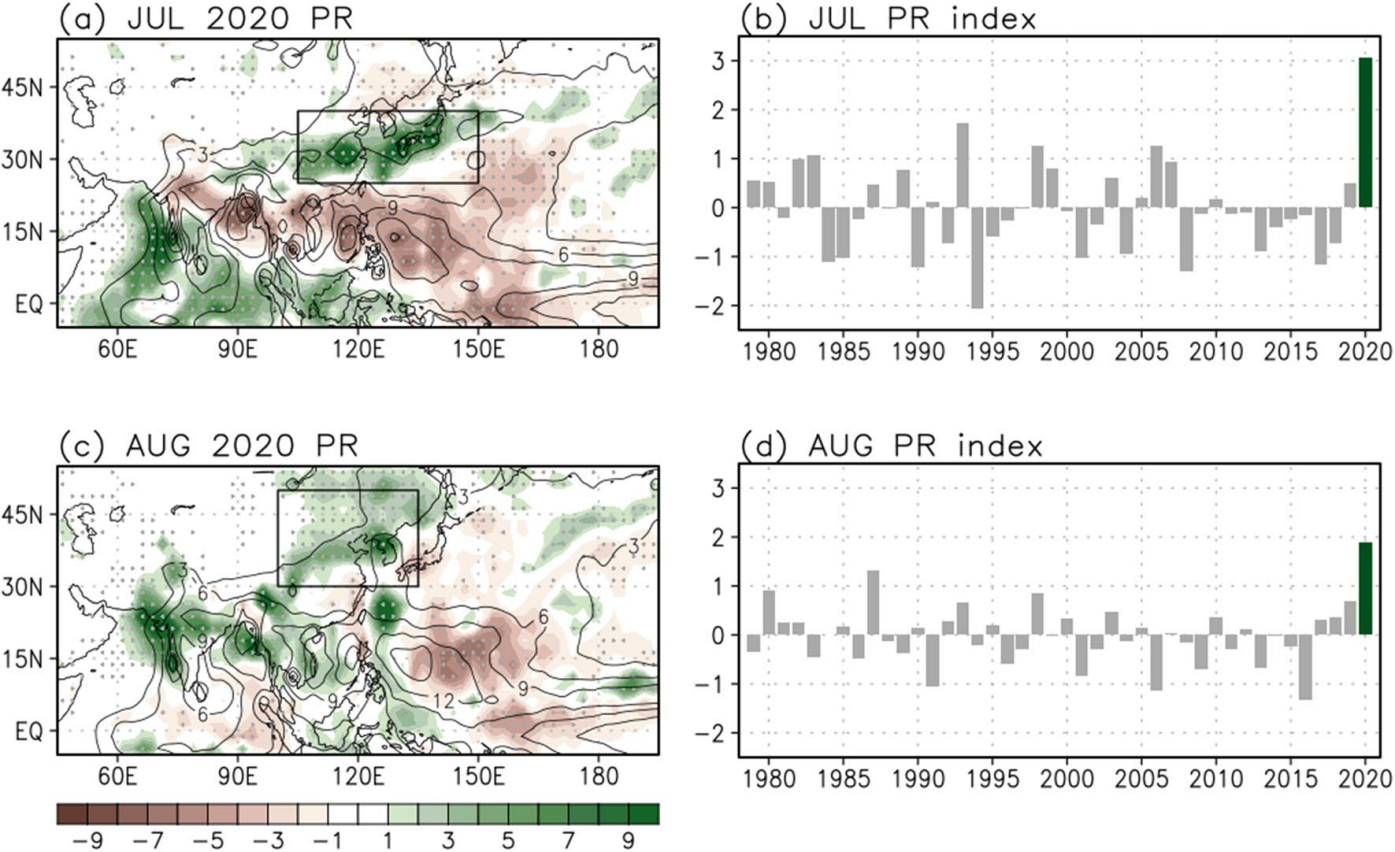
Rainfall climatology (contour; mm/day) in (**a**) July and (**c**) August, and anomalies (shading) in 2020. Values over the 1 standard deviation are stippled. The East Asian regions for July (105°–150° E, 25°–40° N) and August (100°–135° E, 30°–50° N) are indicated by the black-outlined rectangles. Time series of the area-averaged rainfall index in East Asia for (**b**) July and (**d**) August during 1979–2020. The rainfall values of 2020 are in green bars.

During the summer of 2020, an extremely heavy rainfall event triggered devastating floods and landslides in the East Asian regions including China, Korea, and Japan, which caused significant socioeconomic and environmental damage. In July 2020, the zonally elongated rainband was prominent in East Asia (Fig. 1a) striking the record-breaking rainfall event (3.07 mm/day; Fig. 1b). In August 2020, relatively widespread rainfall in East Asia, including eastern Mongolia, northeastern China, and Korea, slightly migrated northward (Fig. 1c) compared to the pattern of July. The rainfall rate in August 2020 was also the largest magnitude since 1979, reaching 1.89 mm/day (Fig. 1d).

Recently, it is suggested that the warm Indian Ocean condition in early summer 2020 traced back to the super Indian Ocean Dipole (IOD) in 2019 could be related to the enhanced rainfall in East Asia during June–July 2020^34,35^. An exceptionally long-lasting and quasistationary Madden–Julian Oscillation (MJO) active phase persisted in the Indian Ocean during June–July 2020 exhibited quite different propagation features apart from traditional MJO events^36^. Superimposed on the tropical Indo-Pacific thermal condition, the MJO activity in June–July 2020 contributed to the rainfall extremes in East Asia, accompanying the low-level anticyclonic anomalies over the WNP that brought abundant moisture to East Asia from the tropics. Additionally, the roles of the MJO and the cooperative influence of the Pacific and Indian Oceans on the rainfall extremes in June–July 2020 were examined^37^.

An extraordinary rainfall evolution recorded in East Asia during June–July 2020 could be linked with a significant quasi-biweekly oscillation (QBWO) and the tropical Indian Ocean as an important external contributor^38^. In addition, the subseasonal phase transition of the North Atlantic Oscillation (NAO) played a key regulator of sequential warm/cold rainband around the Yangtze River from mid-June to mid-July 2020^39^. The origin of the systematically underestimated rainfall anomalies in East Asia for the averaged from July to August 2020 in operational forecasts is examined^40^. The East Asian rainfall anomalies were successfully predicted in the operational seasonal forecast system by correcting the tropical SST forcing applied over the Indian Ocean and tropical central-eastern Pacific.

The results of previous studies suggested that the interbasin SSTs and multiple timescale variabilities play important roles in affecting the record-breaking rainfall extremes in East Asia during summer 2020. Particularly, the East Asian rainfall anomalies and their dynamical causes have been extensively examined in the early summer period (June and July), but possible differences in late summer (August) have drawn little attention. The EASM system can evolve through several subseasonal evolutions throughout the summer season due to strong subseasonal dependency. In the summer of 2020, South Korea experienced a series of intensive rainfall that could be modulated by the combined effects of the anomalous WNP anticyclone via the IPOC effect and summer NAO-related teleconnections^41^. Interestingly, the extreme rainfalls across South Korea in summer 2020 showed different features with a quite sudden transition between two periods (late June to late July and late July to mid-August). As shown in Fig. 1a,b, the detailed patterns of rainfall between July and August 2020 exhibit some differences in its magnitude, center, and extent implying different mechanisms. Therefore, the extraordinary rainfall event in East Asia during August 2020 generated by tropical forcing is an important motivation for the present study.

In this study, the summer rainfall anomalies in East Asia, particularly during July and August, are investigated with a focus on its large-scale dynamics associated with the tropical origins among the Indian, Pacific, and Atlantic Ocean and their interbasin interactions. Two possible key factors are suggested here: tropical Indian Ocean SST during June, and rainfall variability in the subtropical WNP during August affected the north tropical Atlantic SST. We then analyze how the tropical Indian Ocean SST and subtropical WNP rainfall affect the summer rainfall in the East Asian region. Based on the comprehensive understanding of the summer rainfall variability in East Asia, the East Asian rainfall extremes in summer 2020 will be addressed.

## Data

The monthly precipitation data obtained from the Climate Prediction Center Merged Analysis of Precipitation (CMAP)^42^ were used. The monthly geopotential height and horizontal wind data were from the National Center for Environmental Prediction-National Energy Research Supercomputing Center of the Department of Energy Reanalysis II (NCEP-DOE R2)^43^. Both datasets have a 2.5° latitude–longitude resolution. The monthly SST data used were from the National Ocean Atmospheric Administration (NOAA) Extended Reconstructed Sea Surface Temperature version 5 (ERSSTv5)^44^ with a horizontal resolution of 2° × 2°. Only the boreal summer (June–August) is considered for the period from 1979 to 2020. Note that the seasonal cycle and linear trend are removed from all data before analysis.

## Methods

A Linear Baroclinic Model (LBM)^45^ is used to obtain a steady atmospheric response to a prescribed diabatic forcing in the subtropical WNP. The LBM in this study is based on primitive equations linearized about the observed monthly basic state during 1979–2020. The model variables have a horizontal resolution of T21 and 20 sigma levels (T21 L20). The time integration is continued for up to 30 days to approach the steady atmospheric response to a prescribed forcing in this method.

## Results

Unusual heavy rainfall, accompanied by severe flooding and landslides, affected millions of people over large parts of the East Asian countries in summer 2020. However, the detailed patterns of rainfall in July and August 2020 exhibit some differences. In July 2020, the observed rainfalls in East Asia around the latitude of 30° N were well above normal and the highest recorded one (Fig. 1a,b), and the area-averaged rainfall anomaly in East Asia (105°–150° E, 25°–40° N) is exceeding 3.27 standard deviation for the 42-year period. Concurrently, a zonally extended rainfall decrease occurred from the Indian subcontinent to subtropical WNP and increased in the Indian Ocean.

A relatively weak but broad rainfall increase in East Asia was the predominant feature in August 2020, evolving further to the north (Fig. 1c) compared to that of July (Fig. 1a). Because of the northward rainband, the different East Asian regions are selected in July and August based on their maximum rainfall. The rainfall magnitude of East Asia (100°–135° E, 30°–50° N) in August 2020 is above 3.0 standard deviation during the 42 years. In comparison with July 2020, the rainfall decrease still remained in the subtropical WNP but extended further north to around Japan in August 2020. In contrast, the rainfall anomalies from the Indian subcontinent to the South China Sea became positives in August 2020.

**Figure 2:**
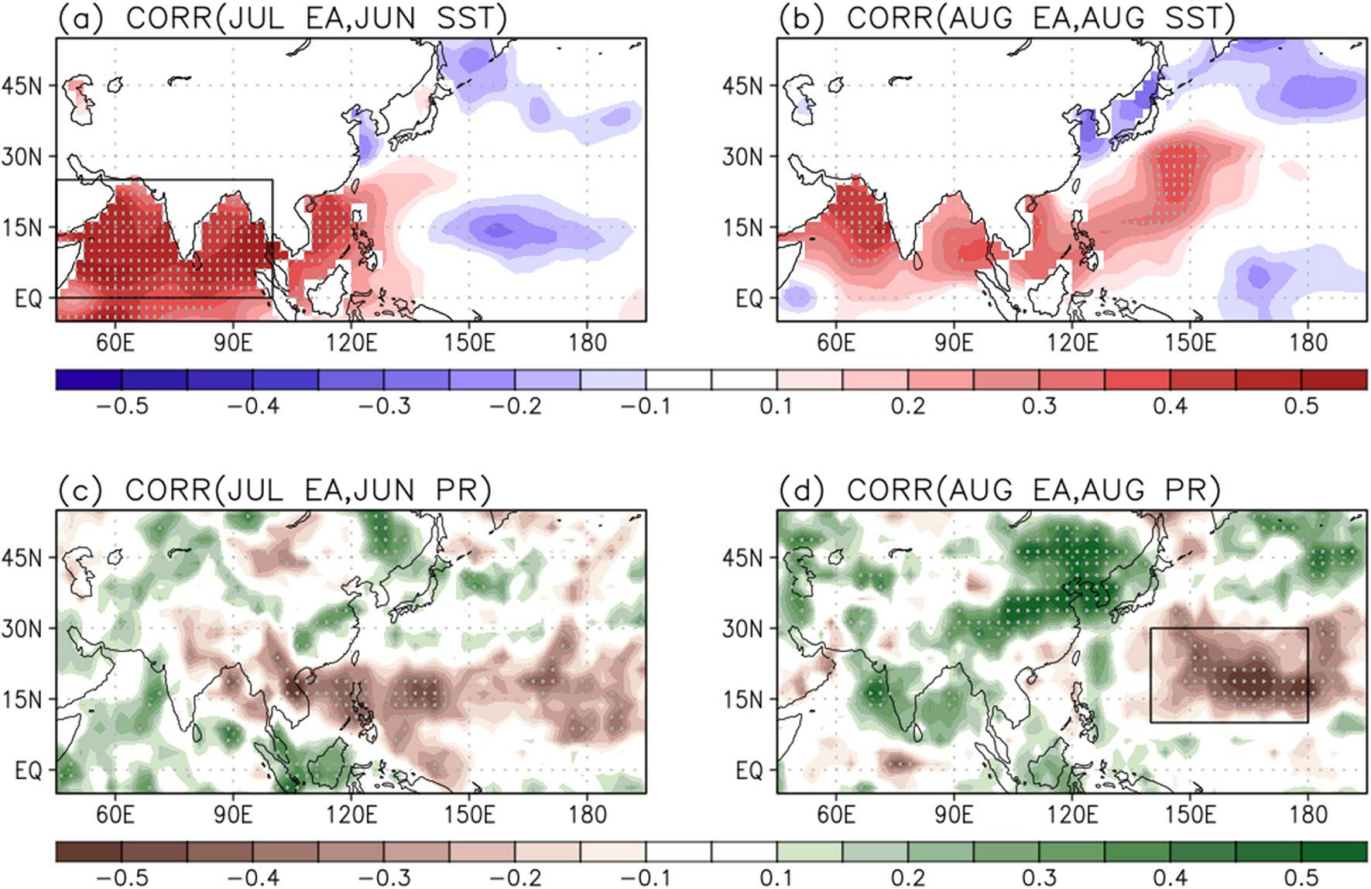
Correlation coefficients of the rainfall index in East Asia (105°–150° E, 25°–40° N) in July for (**a**) SST and (**c**) rainfall anomalies in June during 1979–2020. The same as in (**b**) and (**d**) but for August and the rainfall index in East Asia (100°–135° E, 30°–50° N) in August. Values over the 95% confidence level based on the student t-test are stippled. The SST anomalies in the tropical Indian Ocean (**a**; 45°–100° E, 0°–25° N) and rainfall anomalies in the subtropical WNP (**d**; 140° E–180°, 10°–30° N) region are indicated by the black-outlined rectangles.

The question remains here, what is responsible for the rainfall extremes in East Asia during July and August? To address this, the SST and rainfall anomalies correlated with the East Asian rainfall indices in July and August are shown in Fig. 2. Recently, the delayed impact of Indian Ocean warming on the East Asian surface temperature variation in summer is suggested^32^. The Indian Ocean warming in June is responsible for significant cooling over the Korea–Japan region that peaks in July with a 1-month delay. From the lead-lag correlation with July East Asian rainfall, positive correlations are evident over most of the Indian Ocean and the South China Sea in June with a maximum center in the Arabian Sea and Bay of Bengal (Fig. 2a). This implies that there is a potential role of Indian Ocean SST during June in driving the rainfall increase in East Asia during July. The correlation field of rainfall has a similar spatial pattern to that of July 2020 (Fig. 1a), appearing as west-to-east elongated rainfall decrease from the eastern Indian Ocean to subtropical central Pacific and increase in the western Indian Ocean, yet only significant around the subtropical WNP and central Pacific (Fig. 2c).

The correlations between the August East Asian rainfall index and Indian Ocean SST are weaker than that of June (Fig. 2a), but still significant in the Arabian Sea (Fig. 2b). The negative SST anomalies in the adjacent region to East Asia may be a result of the increased rainfall by reducing shortwave radiation, rather than a cause. Interestingly, the East Asian rainfall anomalies in August are highly correlated with the negative rainfall anomalies in the subtropical WNP (Fig. 2d). In particular, the WNP rainfall decrease was evident in August 2020 (Fig. 1c), suggesting an important role in modulating the East Asian rainfall increase.

As shown in Fig. 2, the highly-correlated factors in potentially explaining the East Asian rainfall are to some extent different for July and August. Given the above results, we choose two possible factors that are responsible for the East Asian summer rainfall: the tropical Indian Ocean SST in June and subtropical WNP rainfall anomalies in August. Therefore, the question arises of how the Indian Ocean SST and WNP rainfall anomalies affect the rainfall variability in East Asia during July and August, respectively. Here, we elucidate the role of two indicators on the development of rainfall in East Asia for July and August.

**Figure 3:**
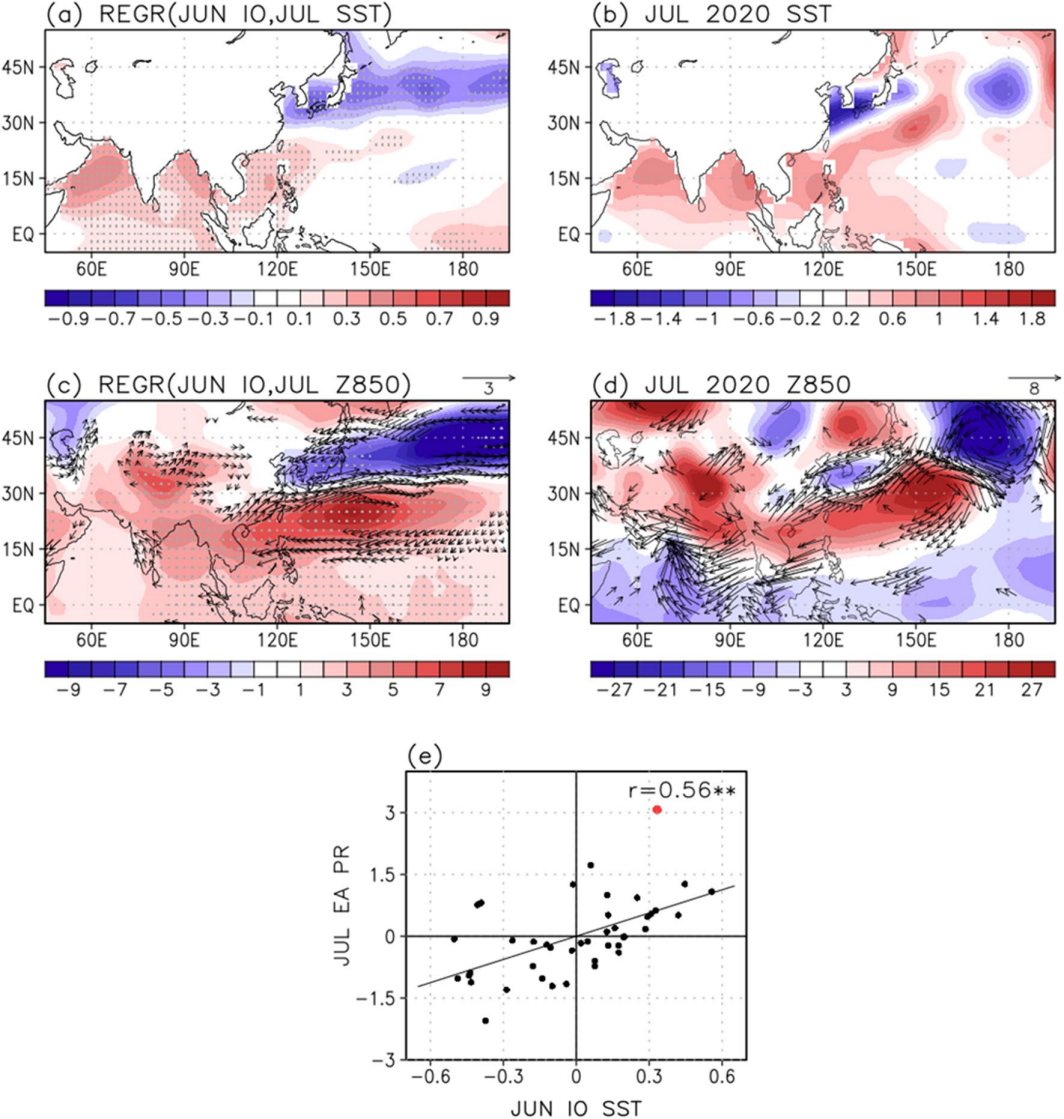
Regressed (**a**) SST (°C), (**c**) geopotential height (shading; m) and wind (vectors; m/s) anomalies at 850 hPa in July onto the Indian Ocean (45°–100° E, 0°–25° N) SST in June during 1979–2020. Note that the regression coefficients are multiplied by the value of Indian Ocean SST in June 2020. Values over the 95% confidence level based on the student t-test are stippled and represented as vectors. (**b**) SST, (**d**) geopotential height and wind anomalies at 850 hPa in July 2020. (**e**) Scatter diagram between the Indian Ocean SST in June and East Asian (105°–150° E, 25°–40° N) rainfall index in July. The value for 2020 is indicated by the red dot. The symbol of ** denotes the 99% confidence level based on the student t-test.

The delayed impact of tropical Indian Ocean SSTs in June on the East Asian rainfall anomalies in July is investigated using regression analysis (Fig. 3). Anomalous SST warming in the Indian Ocean, centered over the Arabian Sea and the eastern Bay of Bengal, is quite similar to that in June, suggesting strong persistency (Fig. 3a). Note that standardized coefficient refers to the regression coefficients simply multiplied by the value of the Indian Ocean SST anomaly in June 2020. In addition, the significant negative SST anomalies are zonally confined near East Asia latitude between 30°–45° N. This regressed SST pattern in July onto 1-month leading Indian Ocean warming (Fig. 3a) is quite similar to the SST anomalies of July 2020 (Fig. 3b), and has a spatial correlation (45° E–165° W, 5° S–55° N) of 0.52.

Traditionally, the tropical Indian Ocean basin warms the season after El Niño^46^, indicating a passive response to El Niño. The relative importance of remote forcing from the Pacific to the Indian sector (i.e., tropical atmospheric bridge)^47^ and internal variability of the Indian Ocean is still debated within the research community^48–52^. The Indian Ocean warming was observed in the summer of 2020 possibly in accordance with the super IOD in fall 2019^34,35^ and/or El Niño event in the winter of 2019/20^53^.

The rainfall responses in July onto the Indian Ocean warming in June clearly show the zonally elongated rainband in East Asia (Supplementary Fig. S1). The Indian Ocean warming enhances convective activity, particularly in the Arabian Sea, because the surface moisture convergence in that region is presumably stronger in comparison with the other Indian Ocean basins^54^. Enhanced convective heating stimulates the tropospheric Kelvin waves that propagate eastward to the western Pacific. Subsequently, the Kelvin wave-induced boundary layer divergence suppresses the local convection in the WNP, and thus low-level anticyclonic anomalies develop through the atmospheric Rossby wave response, namely the IPOC mode^23^.

The resultant low-level anticyclonic anomalies in the WNP are clearly seen (Fig. 3c) as a result of the Indian Ocean warming^22,23^ and are a part of the meridional wave train propagating northward, the PJ teleconnection pattern^14^. Associated with the southwesterlies on the western side of the WNP anticyclonic circulation, large amounts of water vapor can be transported from the tropics to East Asia. Based on the moisture budget analysis, the dynamic effect due to changes in the atmospheric circulation is dominant in July (not shown). Therefore, the Indian Ocean warming in June may be responsible for the positive rainfall anomalies in East Asia in July, exhibiting a 1-month leading role. Note that the Indian Ocean warming was also observed in July 2020 (Fig. 3b), indicating a persistent SST which continuously plays a role in decreasing the WNP rainfall. The low-level atmospheric circulation anomalies in July 2020 (Fig. 3d) resemble the regressed pattern onto the June Indian Ocean warming (Fig. 3c), and the spatial correlation coefficient with geopotential height at 850 hPa is 0.66. The temporal correlation of the Indian Ocean SST during June and the East Asian rainfall anomalies during July is also strong (0.56; Fig. 3e). Importantly, the magnitude of the Indian Ocean SST in June 2020 ranks among the top four since 1979. These results imply that the tropical Indian Ocean warming in June is one of the dominant factors leading to the rainfall extremes in East Asia during July 2020.

**Figure 4:**
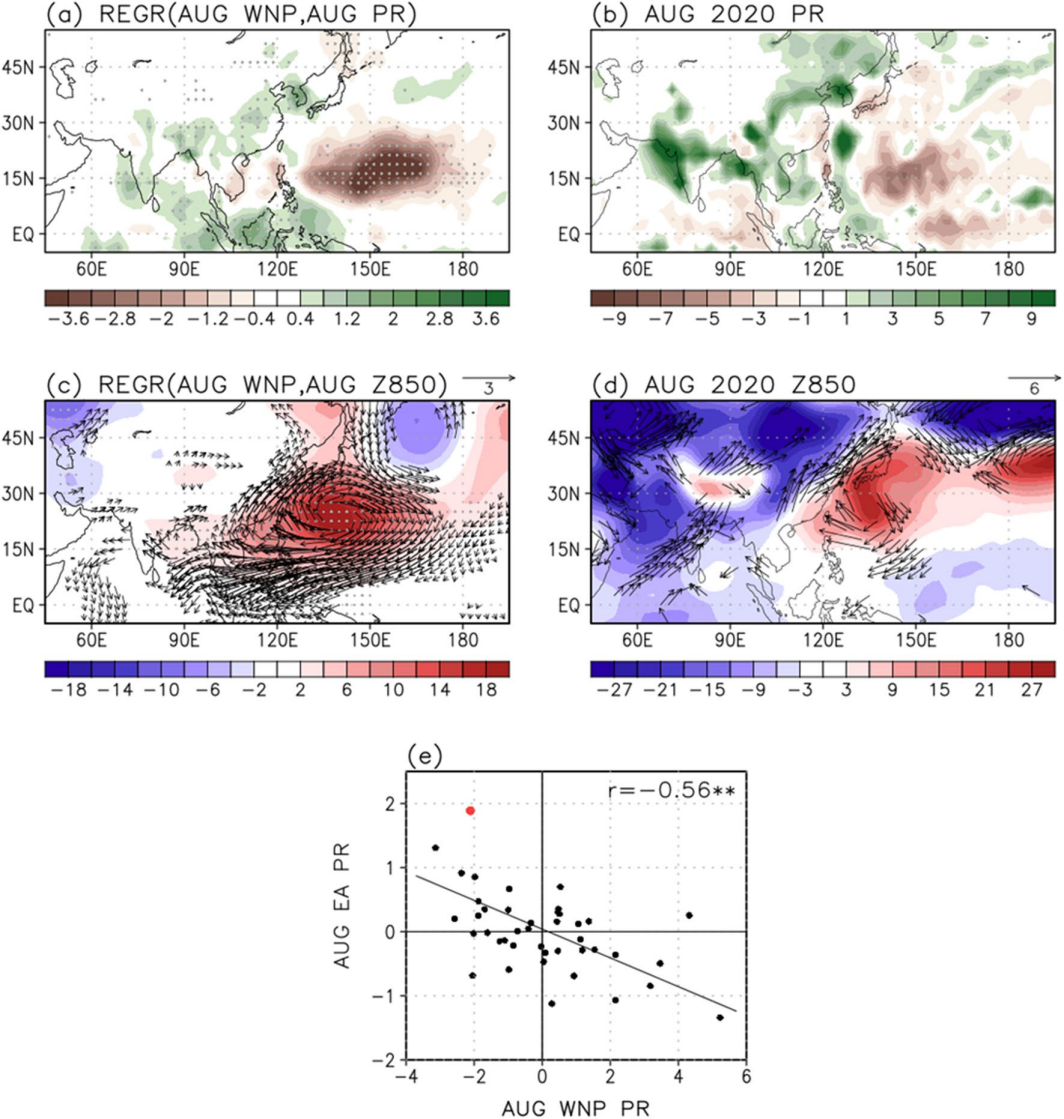
Regressed (**a**) rainfall (mm/day), (**c**) geopotential height (shading; m) and wind (vectors; m/s) anomalies at 850 hPa onto the subtropical WNP (140° E–180°, 10°–30° N) rainfall in August during 1979–2020. Note that the regression coefficients are multiplied by the value of subtropical WNP rainfall in August 2020. Values over the 95% confidence level based on the student t-test are stippled and represented as vectors. (**b**) Rainfall, (**d**) geopotential height and wind anomalies at 850 hPa in August 2020. (**e**) Scatter diagram between the subtropical WNP and East Asian (100°–135° E, 30°–50° N) rainfall index in August. The value for 2020 is indicated by the red dot. The symbol of ** denotes the 99% confidence level based on the student t-test.

In August, associated with the subtropical WNP rainfall index the negative local rainfall anomalies are dominant (Fig. 4a). Concurrently, pronounced rainfall increases in East Asia due to the southwesterlies corresponding to anticyclonic anomalies (Fig. 4c). Although the amplitude of regressed rainfall anomalies in East Asia is weaker than that during August 2020 (Fig. 4b), the signal is statistically significant. Importantly, a rainfall decrease was also evident in the subtropical WNP during August 2020 (Fig. 4b). This indicates that the subtropical WNP rainfall anomalies can play an important role in increasing rainfall in East Asia during August. The simultaneous impact of Indian Ocean SST on the East Asian rainfall in August is relatively weaker than that of Indian Ocean SST in July (0.41) with a correlation coefficient of 0.34, but possibly contributes to the East Asian region.

In response to the subtropical WNP rainfall forcing in August, the anticyclonic anomalies centered at 137.5° E, 22.5° N (Fig. 4c) can be interpreted as a Rossby wave response. The subtropical diabatic forcing in August is far from the equator, thus the wave response can be established at a relatively higher latitude. The resultant anticyclonic flow is accompanied by southwesterly anomalies from the off-equatorial towards East Asia and contributes to the rainfall anomalies there in August. Overall, the atmospheric pattern in August 2020 is quite similar to the regressed result (Fig. 4c), with anticyclonic anomalies evidently centered south of Japan (Fig. 4d). The spatial correlation coefficient between these low-level patterns represented as the geopotential heights at 850 hPa is 0.66. The distribution of subtropical WNP rainfall and East Asian rainfall anomalies for the period 1979–2020 also indicates a significant relationship with a correlation coefficient of − 0.56 (Fig. 4e). In August 2020, the magnitude of the subtropical WNP rainfall anomaly ranks 4th over the last 42 years. This implies that in August the subtropical WNP rainfall decrease may contribute to the development of anticyclonic anomalies, affecting the extreme East Asian rainfall.

**Figure 5:**
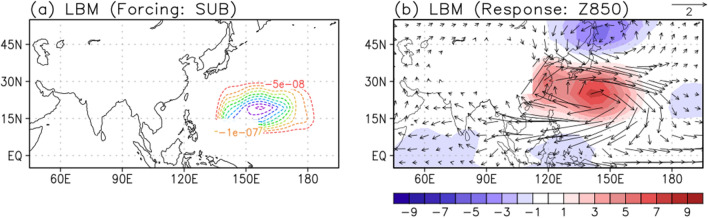
(**a**) The subtropical WNP (140° E–180°, 10°–30° N) diabatic forcing for LBM simulation based on the regressed rainfall pattern in Fig. 4a. (**b**) The geopotential height (shading; m) and wind (vector; m/s) anomalies of LBM simulation in August at 850 hPa during 1979–2020 for the forcing in the (**a**).

To examine atmospheric response to the subtropical diabatic forcing, the LBM^45^ experiment is carried out (Fig. 5). In this experiment, the prescribed forcing is obtained from the linearly regressed local rainfall with respect to the subtropical WNP rainfall index in August (Fig. 4a), using the August basic state during 1979–2020. It is evident that the negative rainfall forcing over the subtropical WNP region (Fig. 5a) is critical in developing the anticyclonic circulation (Fig. 5b), matching well the observational pattern (Fig. 4c). Therefore, the subtropical WNP anticyclonic anomalies can be explained by the negative local rainfall anomalies through the atmospheric Rossby wave response, and then play an important role in modulating the East Asian rainfall in August.

Importantly, the rainfall extremes in July and August 2020 showed different features in East Asia, as a zonally-elongated rainband located near 30° N in July (Fig. 1a), and a relatively broad one confined further north of 30° N in August (Fig. 1c). At the same time, the dominant negative rainfall anomalies over the WNP region are also distinct in July and August, showing that it moves further northeastward in accordance with the northward shifted rainfall in East Asia during August (Fig. 2d), compared to the pattern of July (Fig. 2c). Both the WNP rainfall anomalies in July and August may play an important role in modulating the rainfall variability in East Asia, respectively, although their magnitude and location are different.

However, it is not certain whether the July WNP rainfall evolves to the August one, or another physical process induces the August rainfall independently. To understand the rainfall variability over the subtropical WNP region in August, its preceding SST and rainfall patterns are investigated (Supplementary Fig. S2). The positive local SST anomalies are dominant in June then persist until July, linking to the subtropical WNP rainfall increase in August. However, in August the local SST correlations turn negatives located near 30° N, which can be regarded as a result of rainfall increase rather than a cause. Interestingly, the north tropical Atlantic (NTA) warming in June is significantly correlated with the subtropical WNP rainfall anomalies in August with 2-month leading role, but the correlations over the NTA region become weaker after June. The NTA warming during the spring can induce the low-level cyclonic flow over the eastern Pacific that in turn triggers the low-level anticyclonic flow over the western Pacific in the following months through a subtropical teleconnection^55^. Therefore, the NTA SST anomalies possibly have an impact on the East Asian summer variability through the WNP anticyclonic anomalies^56^.

**Figure 6:**
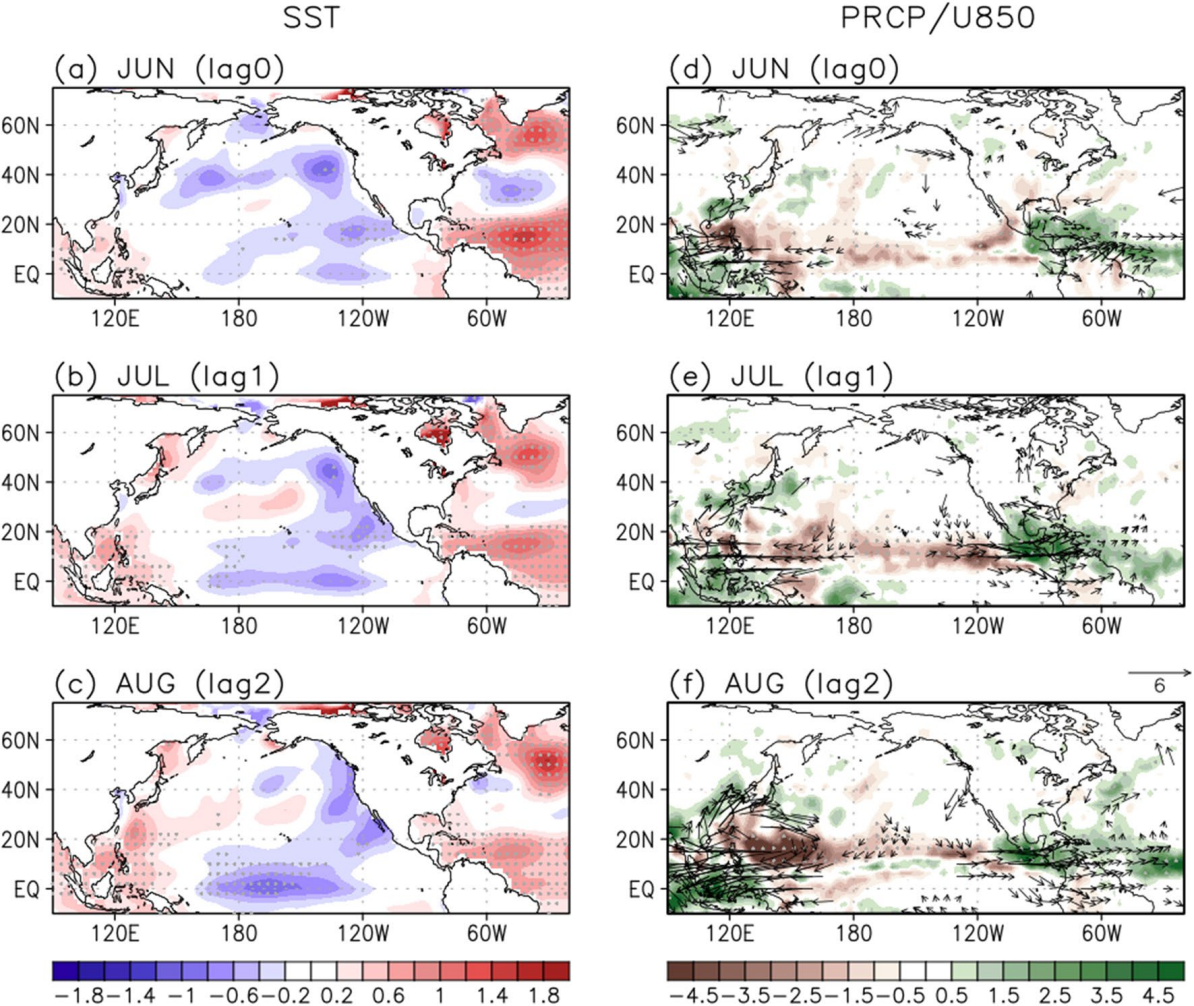
Regressed (left) SST (°C), (right) rainfall (shading; mm/day) and wind (vectors; m/s) anomalies at 850 hPa from June to August onto the NTA (50°–15° W, 0°–20° N) SST in June during 1979–2020, respectively. Values over the 95% confidence level based on the student t-test are stippled and represented as vectors.

The simultaneous and lagged regressions of SST, rainfall, and wind anomalies at 850 hPa onto the NTA (50°–15° W, 0°–20° N) SST anomalies in June are shown in Fig. 6. In June, the positive SST anomalies in the NTA region (Fig. 6a) enhance the local convective activity (Fig. 6d). The resultant diabatic heating gives rise to the low-level cyclonic anomalies over the subtropical eastern Pacific in July as a Gill-type response, with the northerly anomalies on its western side (Fig. 6e). In July, the zonally-elongated rainband in East Asia is quite similar to the observed July 2020 (Fig. 1a), suggesting the potential role of NTA SST anomalies. The northerlies in the subtropical Pacific induce surface cooling through the enhanced wind speed and cold/dry advection from higher latitudes. As a result, the negative rainfall anomalies in the subtropical Pacific induce the subtropical WNP anticyclonic flow until August through the strong air-sea coupling (Fig. 6f). The associated rainfall decrease and anticyclonic circulation in the subtropical WNP region possibly affect the summer rainfall in East Asia during August. The NTA-regressed rainfall increase in East Asia during August is significant (Fig. 6f) and quite similar to that of August 2020 (Fig. 1c). This implies that the NTA warming in June can play a role in modulating the East Asian rainfall in August through the development of subtropical WNP anticyclonic anomalies with 2-month time lag. In June 2020, the NTA region anomalously warmed that is above 0.92 standard deviation during 1979–2020. Recently, the similar results were suggested that the SST anomalies in all three oceans including the Pacific, Indian, and Atlantic Oceans possibly contributed to the record-breaking rainfall that occurred over South China in June 2020, particularly emphasizing a dominant role of Atlantic SST anomalies^57^. Although they focused on the June rainfall condition over South China, the NTA SST anomalies can be a possible precursor of East Asian rainfall anomalies in August.

Additionally, a relatively stronger mean and variability of rainfall exist in the subtropical WNP region during August compared to that during July (not shown) due to the northward migration of the Pacific Intertropical Convergence Zone (ITCZ). Therefore, the interannual variability of subtropical WNP rainfall may well be sensitive to the relatively enhanced climatological local rainfall from July to August. It provides favorable conditions such that enhancement of convective instability in the subtropical WNP extends further north during August. As a result, this strengthened rainfall variability in the subtropical WNP during August may play a more dominant role in modulating local anticyclonic anomalies and have a profound impact on the East Asian rainfall anomalies.

## Summary and discussion

In the 2020 summer, extraordinary rainfall extremes hit East Asia. We show that the tropical Indian Ocean SST and subtropical WNP rainfall anomalies can indeed be responsible for the East Asian rainfall anomalies during July and August, respectively. The Indian Ocean basin warming through summer 2020, extensively considered as a result of super IOD in fall 2019 and El Niño event in preceding winter, possibly modulated the anticyclonic circulation anomalies in the WNP^23^. The WNP anticyclonic anomalies induce the poleward propagating wave train pattern^14^, then the southwesterly anomalies in between the meridional atmospheric dipoles resulted in the rainfall increase in East Asia during July 2020. In August 2020, the widespread rainfall increase in the East Asian region was potentially explained by the southwesterlies associated with the subtropical WNP anticyclonic anomalies, interpreting as a Rossby wave response to the negative local rainfall anomalies. The NTA warming in summer 2020 might be responsible for the development of subtropical WNP anticyclonic anomalies in August 2020, through a subtropical Pacific teleconnection^40^. Additionally, due to the northward migration of Pacific ITCZ, the interannual variability of subtropical WNP rainfall is more favorable conditions that enhanced convection extends further north in August compared to that of July.

It remains an outstanding challenge to predict summer rainfall variability in East Asia, mainly because of the complex climate interaction among signals originating from both the tropics and extratropics. In this study, using reanalysis data the impacts of the tropical Indian Ocean SST and subtropical WNP rainfall forcing on the summer rainfall development in East Asia are investigated. Although the influence of tropical and subtropical originating teleconnections on the East Asian rainfall anomalies are evident here, there may exist other climate factors that contributed to the rainfall extremes in East Asia during summer 2020. In the temporal relationships of tropical forcing and East Asian rainfall anomalies, the anomaly of 2020 lies far away from the linear regression line (red dot in Figs. 3e, 4e). The tropical Indian Ocean SST in June and subtropical WNP rainfall anomalies in August can potentially explain about 31% of the rainfall variability in East Asia during July and August, respectively.

The relative roles of tropical and extratropical factors in different stages of the East Asian rainfall evolution in summer 2020 were suggested^53^. The development of La Niña also could be responsible for the enhanced WNP anticyclonic flow and East Asian rainfalls^10,58^. The relatively weak cooling in the equatorial central Pacific in August 2020 (Supplementary Fig. S1) might contribute to the negative local rainfall anomalies (Fig. 1c), leading to anticyclonic anomalies to its west and depressed convection over the subtropical WNP^59^. A wave train related to the NAO emerges downstream along with the polar-front jet and then modulates the summer rainfall variability around the Yangtze River in summer 2020^39^. In addition, the rainfall increase over India (Fig. 1c) associated with the SST warming in the Arabian Sea possibly excited Rossby wave propagation towards East Asia, known as the circumglobal teleconnection (CGT) pattern^60^. Therefore, relative roles of the tropical, extratropical, and polar origins in the East Asian summer climate will be further pursued.

**Figure 7:**
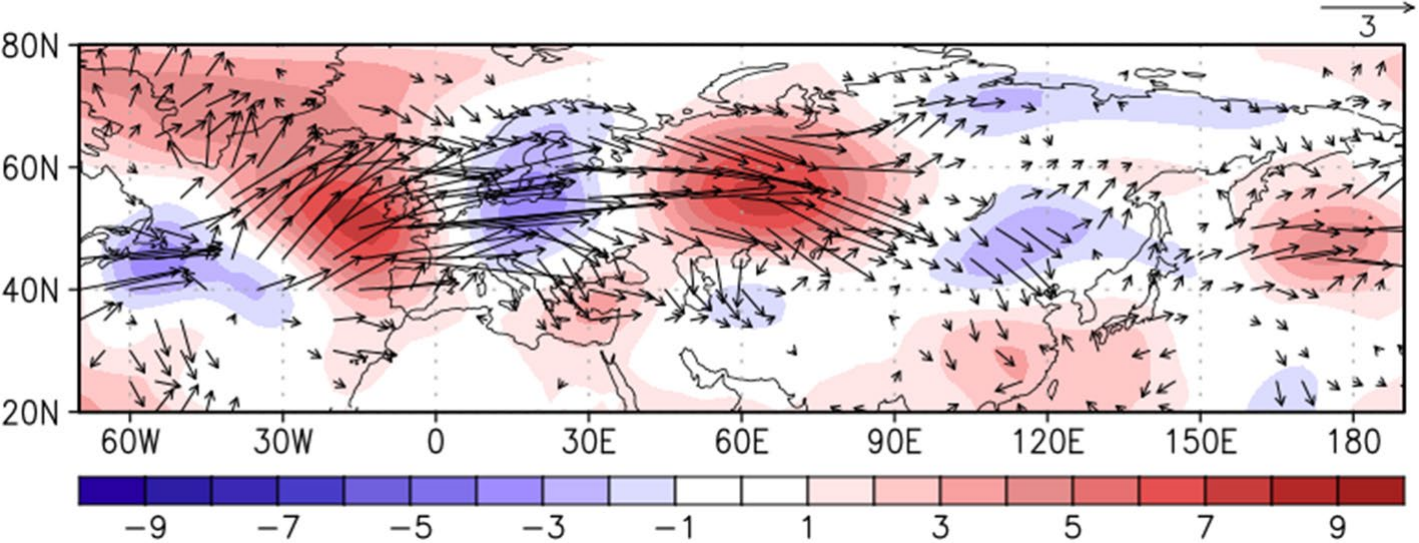
Regressed streamfunction (shading; ${10}^{6}{\mathrm{m}}^{2}\; {\mathrm{s}}^{-2}$) anomalies at 300 hPa and related wave activity flux (vectors; ${\mathrm{m}}^{2}\; {\mathrm{s}}^{-2}$) onto the NTA (50°–15° W, 0°–20° N) SST in August, during 1979–2020. The vectors less than 0.1 ${\mathrm{m}}^{2}\; {\mathrm{s}}^{-2}$ are omitted.

The LBM experiment shows that the subtropical WNP anticyclonic anomalies prescribed by the local diabatic forcing in August partly contribute to the East Asian rainfall anomalies in August. However, the subtropical WNP and East Asian rainfall anomalies can be the results of the same local atmospheric forcing. It is necessary to investigate with coupled models to better understand the causality, mechanism of the teleconnection, and to quantify its impact. The subtropical WNP anticyclonic anomalies in August 2020 extended further northward compared to the regressed atmospheric pattern in Fig. 4c, due to the possible impact of negative precipitation anomalies around Japan (Fig. 4d). The strong cyclonic anomalies also located over northeastern China in August 2020 which could affect the heavy rainfall in the East Asian countries accompanying the southwesterlies in its southeastern part. To examine the pathway of NTA SST anomalies through the extratropical Rossby wave propagation, the wave activity flux at 300 hPa originating from the NTA SST anomalies is shown in Fig. 7. In August, the wave train structure related to the NTA SST anomalies penetrates northern Eurasia and continues to propagate into East Asia. The resultant anticyclonic anomalies are formed in East Asia, partly supporting the development of rainfall anomalies in August. In this study, the impact of NTA SST anomalies on the East Asian rainfall anomalies is mainly suggested through a subtropical teleconnection. However, the pathway of NTA SST anomalies through the extratropical Rossby wave propagation could also have an important role in modulating the subtropical WNP anticyclonic anomalies and the associated rainfall anomalies in August 2020. Therefore, further investigation on the role of the mid-latitude anomalies in August 2020 will be needed.

Under global warming scenarios, summer rainfall in the East Asian region is projected to likely increase^61–65^. However, uncertainties in the projections of future rainfall changes in East Asia among climate models are also large^66–68^. Therefore, understanding the future changes of two possible teleconnection pathways, from the tropical Indian Ocean and subtropical WNP regions, is of great importance for regional rainfall variability in East Asia during summer. Model experiments incorporating future climate change will also provide an assessment of the future subseasonal rainfall variability in East Asia associated with the tropical Indian Ocean SST and subtropical WNP rainfall forcings.

## Supplementary Information

Supplementary Figures.

## Data Availability

The monthly rainfall data were provided by the Center for Climate Prediction Merged Analysis of Rainfall (CMAP), from their website at https://psl.noaa.gov/data/gridded/data.cmap.html. The monthly atmospheric variables by the National Center for Environmental Prediction-National Energy Research Supercomputing Center of the Department of Energy Reanalysis II (NCEP-DOE R2) were obtained from https://psl.noaa.gov/data/gridded/data.ncep.reanalysis2.html. The monthly SST data used from the National Ocean Atmospheric Administration (NOAA) Extended Reconstructed Sea Surface Temperature version 5 (ERSSTv5) were available from https://psl.noaa.gov/data/gridded/data.noaa.ersst.v5.html.

## Code availability

LBM package can be accessed at https://ccsr.aori.u-tokyo.ac.jp/~lbm/sub/lbm.html. All analyses and figures used in this study were performed using PGI Fortran (https://www.pgroup.com/index.htm) and GrADS (http://cola.gmu.edu/grads/). The analysis codes are available on reasonable request.
