# Supplementary material for: Tropical origins of the record-breaking 2020 summer rainfall extremes in East Asia
Source: Sci Rep. 2022 Mar 30;12:5366. doi: 10.1038/s41598-022-09297-4 (PMC8967889; doi:10.1038/s41598-022-09297-4)
Supplement: Supplementary file 1 — Supplementary Figures. [file 41598_2022_9297_MOESM1_ESM.pdf]

Supplementary Information for

Tropical Origins of the Record-breaking 2020 Summer Rainfall Extremes in East Asia

Sunyong Kim[1,2], Jae-Heung Park[2], and Jong-Seong Kug[2]

[1]Physical Oceanography Department, Woods Hole Oceanographic Institution, Falmouth, United States

[2]Division of Environmental Science and Engineering, Pohang University of Science and Technology (POSTECH), Pohang, South Korea

This PDF file includes:

Figure S1 to S2

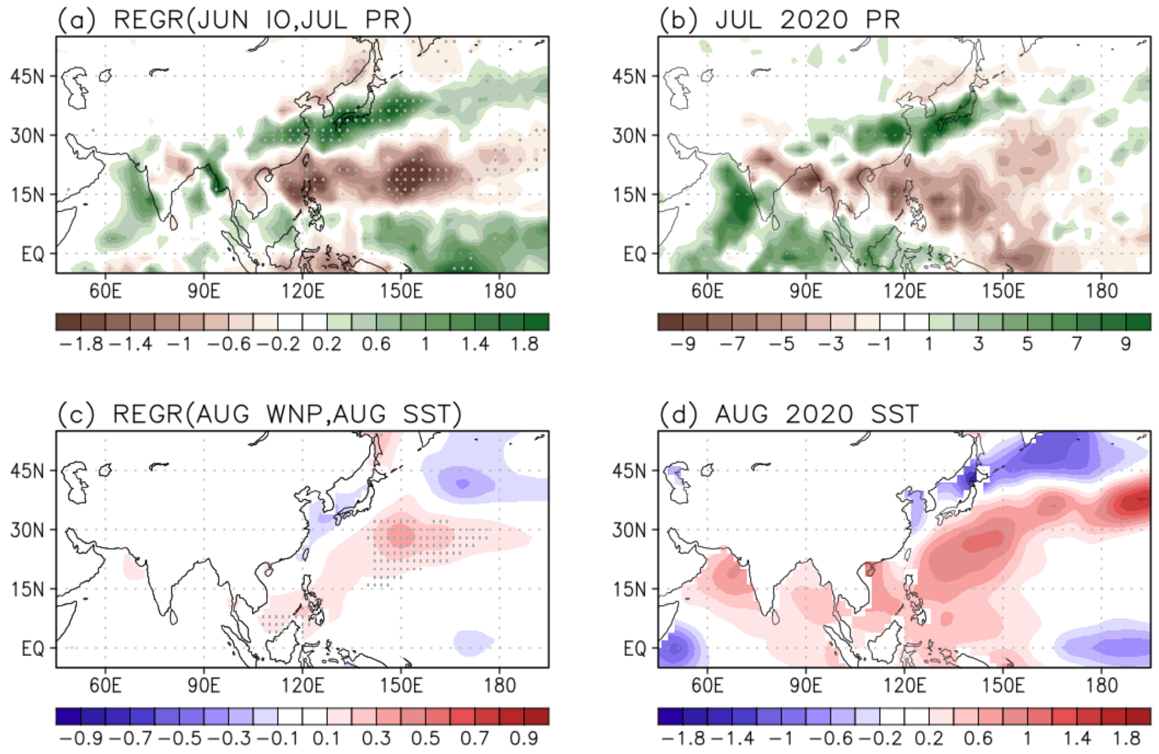

Figure S1. Regressed (a) rainfall (mm/day) in July onto the Indian Ocean (45°-100°E, 0°-25°N) SST in June, and regressed (c) SST (°C) in August onto the subtropical WNP (140°E-180°, 10°-30°N) rainfall in August during 1979-2020. Note that the regression coefficients are multiplied by the value of Indian Ocean SST in June 2020 and subtropical WNP rainfall in August 2020, respectively. Values over the 95% confidence level based on the student t-test are stippled. (b) Rainfall in July 2020 and (d) SST anomalies in August 2020.

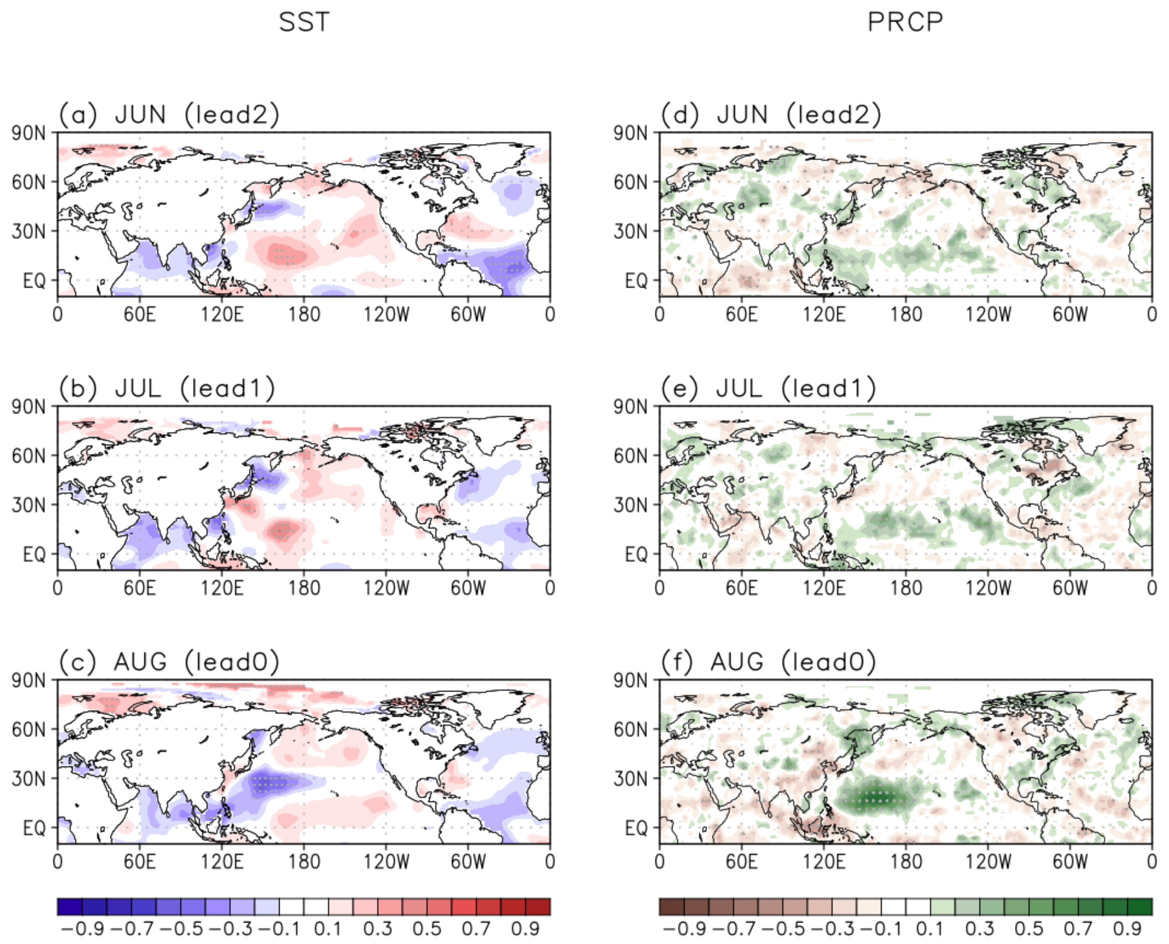

Figure S2. Correlation coefficients of the subtropical WNP rainfall anomalies in August and (left) SST, (right) rainfall anomalies from June to August during 1979-2020, respectively. Values over the 95% confidence level based on the student t-test are stippled.
